# A study of the psychological mechanisms of job burnout: implications of person–job fit and person–organization fit

**DOI:** 10.3389/fpsyg.2024.1351032

**Published:** 2024-08-02

**Authors:** Panpan Zeng, Xiaoli Hu

**Affiliations:** ^1^School of Business Administration, South China University of Technology, Guangzhou, China; ^2^School of Civil Engineering and Architecture, Guizhou Minzu University, Guiyang, China; ^3^Management College, Zhongkai University of Agriculture and Engineering, Guangzhou, China

**Keywords:** person-organization fit, person-job fit, work pressure, job burnout, person-environment fit theory

## Abstract

Job burnout and work pressure are pivotal concerns in human resource management and workplace mental health, profoundly impacting organizational sustainability and individual well-being. Grounded in the person–environment fit theory, this empirical study quantitatively investigates the psychological mechanisms of person–job fit and person–organization fit in job burnout, highlighting the mediating role of work pressure. To test our hypotheses, we investigated 477 employees from 63 IT enterprises around China’s Pearl River Delta region. The findings reveal that person–job fit is negatively associated with job burnout and work pressure, while work pressure positively influences job burnout, partially mediating the relationship between person–job fit and job burnout. Similarly, person–organization fit negatively affects job burnout and work pressure. However, its direct influence on job burnout is insignificant, indicating that work pressure fully mediates the relationship between person–organization fit and job burnout. These findings are consistent with the person–environment fit theory, enhancing our understanding of how individuals fit with their jobs and how organizations affect job burnout through work pressure. This study offers valuable insights for organizations seeking to mitigate burnout and promote employee well-being.

## Introduction

With the rapid development of globalization and escalating market competition, employees’ prolonged emotional involvement and sustained intense work can lead to excessive use of psychological and emotional resources, culminating in job burnout (Maslach et al., 2001). This state of physical and mental depletion significantly hampers organizational growth and employees’ well-being, characterized by increased employee turnover, compromised health, diminished performance, lower organizational citizenship behaviors, and reduced overall well-being (Wang et al., 2015; Turner et al., 2017). Addressing job burnout is crucial not only for organizational advancement but also for safeguarding employees’ occupational safety and mental health.

Academic research has explored the mechanisms of job burnout extensively. Previous studies have primarily focused on individual factors, including personal cognition (such as sense of control, organizational fairness, and values) and personal traits (such as the Big Five personality, self-esteem, and emotional intelligence) (Hu et al., 2017; Kalus and Cregan, 2017; Konstantinou et al., 2018). These studies have focused on the influence of individual factors on job burnout. In addition, the impact of environmental factors on job burnout has received extensive attention from researchers. For example, research has investigated job characteristics, organizational characteristics, and the role of conflict as environmental contributors (Cofer et al., 2018). Recognizing job burnout as more of a social than an individual phenomenon (Maslach et al., 2001), researchers need to consider both environmental and individual factors (Demerouti et al., 2012), delving into their interconnections to fully comprehend their antecedents (Maslach and Leiter, 2008). However, few studies have explored the psychological mechanisms of job burnout from the perspective of person–environment fit (Maslach et al., 2001).

This study integrates the personal and environmental factors of job burnout in a model based on the degree of fit. Person–environment fit consists of five dimensions: person–job fit, person–organization fit, person–vocation fit, person–group fit, and person–supervisor fit (Kristof-Brown et al., 2005). With more in-depth research on the topic of person–environment fit, attention has gradually shifted to the effects of person–job fit and person–organization fit, which have a significant impact on job burnout.

Job burnout has an influence that cannot be ignored. A lack of fit between job needs and employees’ abilities can lead to work pressure, and high workload and time pressure are highly correlated with work pressure and job burnout (Edwards, 1996). The better the person–organization fit, the stronger the employee organizational commitment and the higher the job satisfaction. Employees are less likely to feel physically and psychologically drained when they have high job satisfaction and are willing to be part of the organization for the long term (Boxx et al., 1991). In summary, empirical research examining the relationship between person–organization fit, person–job fit, and job burnout needs to be strengthened to enrich the job burnout literature (Edwards and Cooper, 1990).

The mediating pathways through which person–job fit and person–organization fit influence job burnout also require further exploration. Kristof-Brown et al. (2005) highlighted a research gap in understanding the relationship between person–job fit, person–organization fit, and work pressure. While previous studies have shown the mediating roles of organizational self-esteem, psychological capital, role conflict, and job satisfaction in the relationship between person–job fit, person–organization fit, and job burnout (Kristof-Brown et al., 2005), the mediating role of work pressure in this relationship remains unexplored. Work pressure is a significant cause of job burnout. Despite the positive impacts of challenging stressors on performance, hindrance stress negatively affects employees’ physical and psychological well-being, job satisfaction, and organizational performance (Lepine et al., 2005). Rafferty et al. (2001) found a positive association between high work pressure and job burnout. Studies have shown that person–organization fit can reduce work pressure. Karasek’s (1979) job demand control theory suggests that high job demands, when paired with limited job control, intensify work pressure. A lack of organizational support, job autonomy, and job control can escalate work pressure, influencing job satisfaction. However, the integration of person–organization fit, person–job fit, work pressure, and job burnout into a single analytical framework has yet to be achieved. Thus, the second objective of this study is to investigate the mediating role of work pressure in the relationship between person–organization fit, person–job fit, and job burnout.

Building on the identified gaps, this study utilizes data from 63 IT companies in the Pearl River Delta to examine the effects of person–job fit and person–organization fit on job burnout, employing work pressure as an additional explanatory mechanism for the relationship between person–organization fit, person–job fit, and job burnout. The study’s theoretical contributions are threefold: first, it expands the exploration of job burnout’s antecedent variables; second, it uncovers the internal mechanism linking person–job fit, person–organization fit, and job burnout; and third, it reveals that person–organization fit does not directly impact job burnout, differing from previous study conclusions.

## Literature review and research hypotheses

### Person–job fit, person–organization fit and job burnout

It is widely recognized in academia that person–job fit refers to the fit between demand and supply and between demand and ability (Cable and Judge, 1996; Tong et al., 2015). This paper focuses on two core dimensions of person–job fit: first, the fit between job demand and individual ability, and second, the fit between employee need and job supply. Maslach and Jackson (1981) conducted an in-depth study of job burnout and summarized three core dimensions: emotional exhaustion, cynicism and low occupational effectiveness. These dimensions have gained wide acceptance in the academic community, and this paper follows this perspective. Emotional exhaustion reflects the depletion of employees’ emotional resources, cynicism reveals their negative attitudes toward their work and the interpersonal relationships it involves, and low occupational efficacy refers to their lowered sense of self-efficacy and negative self-assessment of occupational achievement (Maslach and Jackson, 1981).

Person–environment fit theory suggests that individual behavioral performance is influenced by the interaction of individual characteristics and the environment in which they are located. It emphasizes the similarity, fit or congruence between individuals and their environments (Kristof-Brown et al., 2005). Based on this theory, this study explores the relationship between person–job fit and job burnout. When there is a lack of fit between job requirements and personal capabilities, employees may feel that their skills, knowledge and experience are insufficient and that they need to work harder to meet the job standards. This lack of fit can lead to long-term tension and the constant depletion of emotional resources, ultimately leading to emotional exhaustion. At the same time, due to difficulties in performing their jobs, employees may gradually lose their enthusiasm for work, become passive and apathetic, and take a cynical approach to their work. Persistent frustration may cause employees to doubt their ability and value, leading to low occupational efficacy.

From the perspective of person–job fit theory, when the fit degree is low, employees have fewer resources and less energy and are more likely to experience job burnout, verifying the negative correlation between person–job fit and job burnout (Zhang, 2022) A lack of fit between job supply and individual needs also affects job burnout. When the job does not fulfil the employee’s needs, they can feel lost. The job is perceived to lack meaning, or the work environment and conditions do not meet their expectations. This lack of fit causes employees to lose interest and motivation, creating emotional exhaustion. At the same time, there is a sense of distrust and alienation from the organization and a cynical work attitude. Unmet long-term needs may leave employees confused about their career prospects, which in turn reduces their sense of career efficacy. Babakus et al. (2011) further showed that person–position fit not only directly affects job burnout but also mediates the relationship between customer orientation and servant leadership and job burnout and turnover intention.

Person–organization fit, which is mainly reflected in the fit between individuals and organizations in terms of values, goals and missions (Kristof-Brown et al., 2005), is a crucial factor in job burnout. First, when individuals and organizations are aligned on values, it reduces employees’ internal conflicts and contradictions at work, decreases the consumption of emotional resources and thus alleviates emotional exhaustion. This increased fit also enhances employees’ organizational commitment and job satisfaction, which in turn reduces the risk of job burnout due to physical and psychological exhaustion (Boxx et al., 1991). Second, in terms of goal fit, a high degree of individual and organizational goal congruence gives employees greater clarity of work direction and can reduce confusion and uncertainty, thus reducing cynicism. Kilroy’s study found that high involvement in work practices was significantly negatively correlated with job demands and burnout (Kilroy et al., 2016).

Finally, in terms of mission fit, when an employee is closely connected to the mission of the organization, they will be more likely to feel the significance and value of their work, which can stimulate enthusiasm for work to improve career efficacy. Verquer et al. (2003) found that person–organization fit is positively correlated with job satisfaction and organizational commitment, and negatively correlated with turnover intention. Xu et al. (2015) used literature analysis to propose that person–organization fit has a negative effect on job burnout and turnover tendency, which provides a powerful analytical framework for subsequent studies. In conclusion, we propose two hypotheses:

*H1*: Person–job fit negatively affects job burnout.

*H2*: Person–organization fit negatively impacts job burnout.

### Person–job fit and work pressure

Work pressure is a dynamic process in which employees experience a series of physiological, psychological and behavioral responses to job demands (stressors) that exceed their coping abilities (resources) (Lepine et al., 2016). Work pressure often arises when there is a lack of fit between individual characteristics and the demands of the work environment (Sell, 1984). First, when employees’ work experience, educational background, personality traits and natural strengths have a low degree of fit with the task requirements of their jobs, they need to invest much time and energy to complete the tasks and make up for the lack of their skills through continuous learning. This lack of fit invariably increases the work pressure on employees. Edwards and Cooper (1990) studied person–environment fit and coping with pressure, and pointed out that a lack of fit between job requirements and employees’ abilities leads to work pressure. When an individual’s ability does not meet the requirements of their job, job performance is affected, and they may experience work pressure (Kristof-Brown et al., 2005).

Second, when the work itself can satisfy employees’ needs in various ways, such as personal sense of achievement and self-worth, they take the initiative to delve into the business and improve their sense of achievement from work, which also reduces the psychological pressure of the job. Finally, person–job fit can positively influence employees’ attitudes and emotions. Employees with a high degree of person–job fit have more knowledge and skills, have confidence in completing their work tasks, and perceive less stress from their work. Chilton et al. (2005) survey of 123 software engineers found that those who had a strong fit with the cognitive style of their jobs experienced the least amount of work stress. The researchers called on future scholars to test the relationship between job fit and work pressure in other occupational fields (Chilton et al., 2005).

Based on these insights, we propose:

*H3*: Person–job fit has a negative effect on work pressure.

### Person–organization fit and work pressure

From the perspective of person–environment fit theory, first, the lack of fit between the employee and the organization may be that the employee’s personal beliefs and values are not in line with what the organization promotes. This may make the employee psychologically uncomfortable, thus increasing work pressure. When there is a large difference in values between the person and the organization, it tends to create negative psychological effects, which in turn creates work pressure (Edwards and Cooper, 1990). In addition, employees who feel that they are not accepted or recognized by the organization may develop feelings of loneliness and alienation, and this emotional stress can also translate into work pressure.

Second, employees whose personal development goals are not aligned with those of the organization may find promotion opportunities limited or have difficulty accessing training and development opportunities. Obstructed career development also translates into work pressure. Nur Iplik et al. (2011) showed that person–organization fit promotes employee communication and access to organizational support, which reduces work pressure. Again, a lack of fit between employees and the organization’s mission may lead to communication barriers. Employees may have difficulty understanding or accepting the organization’s decisions and actions, and the organization may have difficulty effectively communicating its mission and goals to employees. This miscommunication can increase misunderstandings and conflicts, which in turn can increase work pressure. Chen and Cunradi (2008) found that person–organization fit affects employee job satisfaction through work pressure in a survey of 225 Beijing restaurant employees.

Therefore, we propose:

*H4*: Person–organization fit has a negative effect on work pressure.

### Work pressure and job burnout

Numerous studies have shown that work pressure is a predictor of job burnout. First, prolonged work stress requires employees to invest a great deal of emotion and energy in coping with work challenges, and this continuous depletion can lead to the gradual depletion of employees’ emotional resources. Emotional depletion occurs when employees can no longer cope with additional work stress. Chen and Cunradi (2008) study of 1,231 bus drivers found a highly positive correlation between daily work stress and job burnout. Second, in a high-pressure work environment, employees may feel that their efforts and dedication are not properly rewarded, and a sense of loss and frustration may cause them to lose enthusiasm and interest and develop a cynical mindset. This may result in indifference to work tasks and indifference and detachment toward coworkers and superiors. Maslach and Leiter (2008) noted that job burnout was a response to chronic work pressure and that high workloads and time pressure were highly correlated with work pressure and job burnout. Finally, when employees are faced with excessive work pressure, they may feel inadequate in their ability to cope with challenges or feel that their work results are not recognized. This sense of frustration and powerlessness leads to a gradual decrease in occupational efficacy. Wang et al. (2015) questionnaire survey of 521 Chinese functionaries found that work pressure was negatively correlated with job satisfaction and positively correlated with job burnout among functionaries. The relationship between work pressure and job satisfaction was mediated by job burnout. A sense of control served as a moderator in the relationship between work pressure and job burnout (Wang et al., 2015). Griffin et al. (2010) concluded that work pressure was positively related to job burnout.

Hence, we propose:

*H5:* Work pressure has a positive effect on job burnout.

**Figure 1:**
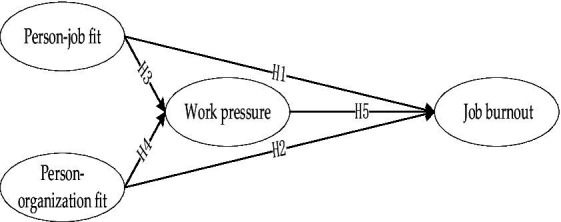
The theoretical model.

The theoretical model for this study is illustrated in Figure 1.

## Research methodology

### Study sample

We followed Cohen’s (1988) recommendations and set the effect size to 0.2, the significance level to 0.05, and the power value to 0.9. The software analysis conducted using G*Power software indicated that our study should collect at least 314 questionnaires to test the proposed hypotheses. To validate the proposed model, we gathered data from employees working in internet enterprises around the Pearl River Delta region in China. We first contacted the career guidance center of the South China University of Technology (SCUT) and the career departments of universities in the Guangzhou area, asking them to provide a list of companies. Next, we randomly selected 63 internet companies in Guangzhou and contacted the chief executives (CEOs) of these enterprises to ask permission for their employees to participate in our study. Their human resource supervisors (HRMs) assisted with the distribution and collection of the questionnaires. Then, we sent the electronic version of the questionnaires to HRMs. We indicated that employees’ participation was voluntary and that their answers were anonymous and would be used for research purposes only, and we informed them they had the right to withdraw from the study at any time. From March to May 2021, 1,260 questionnaires were distributed to employees, and 505 were returned. After 28 were excluded for having 4 or more missing items, 477 questionnaires were used for hypothesis testing.

### Measurement of variables

For variable measurement, the study utilized well-established scales in the field, translated into Chinese using a double-blind method. All scales were measured on a 5-point Likert-type scale.

1. Person–organization fit was measured using the Cable and Judge (1996) 7-item scale. Three sample items are: the company’s values and my values are similar; I feel that my personality traits and the company’s image traits are a good fit; The company can meet my needs. The internal consistency coefficient of the person–organization fit scale was 0.95, indicating good internal consistency.
2. Person–job fit was measured using Weng’s (2010) 4-item scale. Three sample items are: I feel that I am a good fit for this job; the requirements of this job are consistent with the experience, skills and knowledge I have; the work environment provided to me by the organization is consistent with my expectations. The internal consistency coefficient of the person–job fit scale was 0.94.
3. Work pressure was measured using Wang‘s three-item scale (Wang and Zhang, 2016). Three sample items are: My job is extremely stressful; there are very few things in my job that are not stressful; I feel a great deal of stress about my profession. The internal consistency coefficient of the scale was 0.85.
4. Job burnout was measured using Li and Shi’s 5-item scale (Li and Shi, 2003). Three sample items are: Work makes me feel physically and mentally exhausted; I feel exhausted when I leave work; I feel very tired when I get up in the morning and have to face the day’s work. The internal consistency coefficient of the scale was 0.92.
5. For control variables, in line with previous studies (e.g., Mumford et al., 2002), gender, age, and education level were identified.

## Data analysis

### Data characteristics

As Table 1 shows, in the final sample, the number of participants from each company ranged from 2 to 25, with an average of 7.57 participants per company. In terms of gender, 53.7% were male and 46.3% were female. The average age was 33.71 years (SD = 4.51) and the average organizational tenure was 2.17 years (SD = 2.11), with 32.7% possessing a college diploma, 59.5% with a bachelor’s degree, and 7.8% with a postgraduate degree.

**Table 1:** Employee characteristic statistics.

Feature Dimension	Category	Frequency	Percent	Value
Gender	Male	256	53.7%	
	Female	221	46.3%	
Age	21–30	250	52.5%	
	31–40	165	34.8%	
	41 and above	12	2.5%	
	Average			33.71 years
	Others	48	10.2%	
Education Level	Associate degree	9	1.8%	
	Bachelor’s degree	150	31.6%	
	Master’s degree	280	58.8%	
	Doctoral degree	38	7.8%	
Working Duration	Minimum			1 month
	Maximum			17 years
	Average			2.16 years
	Standard deviation			2.11 years

### Common method variance testing

Although the participants’ anonymity was assured, all the data were self-reported from a single source. Thus, we cannot ignore the potential common method variance. Harman’s one-factor test was used to address the issue of common method variance. The results showed that the first common factor explained 39% of the variance of the total variance without rotation, which did not exceed half of 82.29%, suggesting that common method variance was not a serious issue.

### Confirmatory factor analysis

Before testing the theoretical model, following the recommendation of Anderson and Gerbing (1988), we performed a series of confirmatory factor analyses for the four variables (person–organization fit, person–job fit, work pressure, job burnout) to assess their construct distinctiveness using Mplus software.

The results are presented in Table 2. The proposed four-factor model fits the data well and offers a significant change in RMSEA (>0.01; Chen, 2007) and CFI (>0.01; Cheung and Rensvold, 2002) over a three-factor model (∆ RMSEA = 0.038, ∆ CFI = 0.066), the two-factor model (∆ RMSEA = 0.137, ∆ CFI = 0.350), the one-factor model (∆ RMSEA = 0.17, ∆ CFI = 0.487), indicating discriminant validity (Cheung and Rensvold, 2002; Chen, 2007).

**Table 2:** Validation factor analysis of measurement scales.

Models	*χ*^2^	df	*χ*^2^/df	RMSEA	CFI	TLI
Four factors	569.591	140	4.069	0.08	0.949	0.938
Three factors	1131.107	149	7.591	0.118	0.883	0.866
Second factor	3532.008	151	23.391	0.217	0.599	0.546
One factor	4683.074	152	30.81	0.25	0.462	0.395

In the one-factor model, all items were loaded on one factor. In the two-factor model, person–job fit, person–organization fit, work pressure and job burnout were loaded on one factor. In the three-factor model, person–job fit and person–organization fit were loaded on one factor. In the four-factor model, person–job fit, person–organization fit, work pressure, job burnout.

### Descriptive statistics

The descriptive statistics, including means, standard deviations and correlation coefficients for the study’s variables, are presented in Table 3. The data were deemed suitable for testing.

**Table 3:** Table of means, variances and correlation coefficients between variables.

	Average Value	Variance	1	2	3	4	5	6	7
GE	1.463	0.499	1						
YB	1989.289	4.510	0.084	1					
AQ	2.732	0.625	−0.133**	−0.213**	1				
PO	3.400	0.822	−0.081	−0.092*	0.094*	1			
PJ	3.546	0.808	−0.100*	−0.153**	−0.01	0.593**	1		
WS	3.185	0.813	−0.117*	−0.138**	0.046	−0.056	0.170**	1	
BOUT	2.734	0.895	−0.107*	−0.056	0.087	−0.212**	−0.075	0.677**	1

GE, gender; YB, year of birth; AQ, academic qualifications; PO, person–organization fit; PJ, person–job fit; WS, work pressure; BOUT, job burnout. *p < 0.05; **p < 0.01.

### Hypothesis testing

We tested the hypotheses by employing structural equation modeling using Mplus 7.0. This approach presents greater statistical performance than regression analysis, a technique that can only examine models one step at a time. Additionally, to obtain more accurate statistical performance, a bootstrap approach with 5,000 bias-corrected bootstrap samples was run, and significance was established with a 95% confidence interval (CI) that excludes zero. All constructs were regarded as latent variables in the model. The results show that the model presents an acceptable fit to the data (*χ*^2^ = 569.591, df = 140 (*p* < 0.001), CFI = 0.949, RMSEA = 0.08), and the path coefficients are shown in Table 4.

**Table 4:** Coefficients of paths for the proposed model.

Paths	Coefficients	95% CI
Person–job fit→job burnout	−0. 239^**^	−0.195, −0.127
Person–job fit→work pressure	−0.358^**^	−0.414, −0.369
Person–organization fit→job burnout	−0.044	−0.076, 0.044
Person–organization fit→work pressure	−0.276^**^	−0.366, −0.320
work pressure→job burnout	0.761^**^	0.797, 0.855

*p < 0.05; **p < 0.01.

As shown in Table 4, person–job fit is negatively related to job burnout (*B* = −0.239, *p* < 0.01, 95% CI [−0.0195, −0.127]) and work pressure (*B* = −0.239, *p* < 0.01, 95% CI [−0.0195, −0.127]). Therefore, Hypotheses 1 and 3 are supported. In contrast, person–organization fit was not significantly related to job burnout (*B* = −0.044, *p* > 0.05, 95% CI [−0.076, 0.044]) but was negatively related to work pressure (*B* = −0.276, *p* < 0.01, 95% CI [−0.366, −0.320]). Therefore, Hypothesis 4 was supported, but Hypothesis 2 was not. For the association between work pressure and job burnout, the results indicated that work pressure was positively related to job burnout (*B* = 0.761, *p* < 0.01, 95% CI [0.797, 0.855]). Taken together, five of the hypotheses in the current study were validated, but the relationship between person−organization fit and job burnout was not indicated by the data. As such, it can be concluded that work pressure plays a partial mediating effect in the relationship between person–job fit and job burnout, and it acts as a full mediator in the relationship between person–organization fit.

## Discussion

In this paper, based on the person–environment fit theory, we constructed and tested a model to explore the psychological mechanisms between person–job fit, person–organization fit and job burnout, focusing on the mediating role of work pressure. The results showed that (1) Hypothesis 1, the negative effect of person–job fit on job burnout, was supported by the data. This explains why person–job fit can reduce job burnout. (2) Hypothesis 3, the negative effect of person–job fit on work pressure, was supported. This is consistent with previous studies (Kristof-Brown et al., 2005). (3) The results of the data analysis support the positive effect of work pressure on job burnout, i.e., Hypothesis 5 is supported. This shows that person–job fit influences job burnout through work pressure. It shows that there is a significant partial mediating effect of work pressure on the effect of person–job fit on job burnout. (4) The results of the data analysis support Hypothesis 4, the negative effect of person–organization fit on work pressure.

However, there is also a new finding in this study: Hypothesis 2, the direct effect of person–organization fit on job burnout, was not supported. This is inconsistent with previous studies (Cable and Judge, 1996). A possible explanation for this is the presence of highly correlated variables in the same model, with person–organization fit and person–job fit often considered highly correlated (Avey et al., 2010). The relationship between job burnout and person–organization fit may be covered by those with person–job fit. Therefore, the findings that person–organization fit affects job burnout through work pressure and that work pressure plays a fully mediating effect have important implications for the development of related theories and business management practices.

### Theoretical implications

This paper contributes to the theoretical and empirical understanding of person–job fit, person–organization fit, work pressure, and job burnout within the context of China’s internet industry. Key aspects include:

- Theoretical extension: This study addresses the hot topic of job burnout, adopting a fit perspective. It integrates environmental and individual factors, along with their interactions, to elucidate job burnout. This approach advances our comprehension of the psychological mechanisms driving job burnout due to person–job and person–organization fit, offering a refined perspective on the influencing factors. This not only broadens the scope of person–environment fit theory but also augments and diversifies the existing body of research on job burnout.
- New insights: Contrary to the widely held belief that person–organization fit directly mitigates job burnout, this research reveals no significant direct impact. This contrasts with earlier studies (Cable and Judge, 1996; Maslach et al., 2001), suggesting that person–organization fit influences job burnout predominantly through work pressure, which acts as a complete mediator. Thus, reducing work pressure may have a more immediate effect on mitigating job burnout than enhancing person–organization fit.
- Work pressure as a mediator: Historically, the role of work pressure as a mediator in the relationship between person–job fit, person–organization fit, and job burnout has been underexplored. This study breaks new ground by examining work pressure as a mediator, finding a significant partial mediating effect of work pressure on the impact of person–job fit on job burnout. It establishes that person–organization fit affects job burnout through work pressure, which exerts a fully mediating effect. These insights pioneer a novel approach to understanding the mechanisms through which person–job fit and person–organization fit influence job burnout.

### Practical implications

This study’s findings have significant managerial implications.

- Individual and Organizational Alignment: Individuals must align both with their job roles and the organizational culture to mitigate job burnout risks. Similarly, organizations must prioritize hiring individuals whose values resonate with the organizational ethos during the staff selection process. Post-hiring, aligning employee competencies with job demands is vital. Investments in human and financial resources, such as thorough selection processes and pre-employment training, are key to enhancing this fit. This study underscores the effectiveness of such investments in reducing job burnout, a connection not extensively supported by prior systematic research.
- Influence of HRM Practices: HRM practices can significantly shape employees’ perceptions of their fit within the organization and their specific roles. These practices, including tailored career development plans aligned with employees’ current positions, can alleviate work pressure and consequent job burnout. Research by Boon et al. (2011) indicates that effective HRM practices enhance the congruence between employee needs and job requirements, leading to improved job satisfaction and reduced job burnout. By assisting employees in selecting suitable roles and adapting to organizational culture, these practices help maintain psychological well-being (Boon et al., 2011).
- Mitigating Job Burnout through Stress Management: Beyond enhancing person–job and person–organization fit, reducing job burnout is achievable by alleviating work pressure. This can be done through effective time management, and personal and organizational stress management strategies and skills. Emerging research shows that time management behaviors can significantly lower work pressure, aiding in the reduction of job burnout (Jex and Elacqua, 1999; Gillespie et al., 2001).

### Limitations and future directions

This study yields several important conclusions, yet four primary limitations should be acknowledged. First, utilizing cross-sectional data to validate our theoretical model curtails our capacity to definitively ascertain causality between the independent and dependent variables. Future research should employ longitudinal data to more accurately assess this. Second, the exclusive participation of IT company employees from China’s Pearl River Delta may limit the broader applicability of our findings. Replication in diverse geographical and cultural contexts could affirm the universality and theoretical relevance of our results. Third, our research focused solely on work pressure as a mediating variable. Future studies should explore additional mediating factors. Notably, the disparity between employee skills and job demands could intensify various stressors, such as diminished self-efficacy, perceived workload, lack of job control and role overload. These stressors may escalate work pressure, potentially culminating in job burnout. Finally, based on the findings reported in existing studies (Khosravi et al., 2020; Khosravi, 2021, 2023), individuals with high levels of novelty-seeking personality traits have been associated with higher levels of job burnout. Incorporating this personality trait in our model as a moderator could generate a more accurate interpretation of future results.

## Data availability statement

The original contributions presented in the study are included in the article/Supplementary material, further inquiries can be directed to the corresponding authors.

## Ethics statement

This study was carried out in accordance with the recommendations of the Ethics Committee of the SCUT. The protocol was approved by the Ethics Committee of the SCUT. All subjects were given written informed consent in accordance with the Declaration of Helsinki” (see page 9). I understand the importance of the Ethics Committee or Institutional Review Board approval for research involving humans or animals. I want to assure you that our manuscript adheres to ethical guidelines. The studies were conducted in accordance with the local legislation and institutional requirements. The participants provided their written informed consent to participate in this study.

## Author contributions

PZ: Writing – original draft, Writing – review & editing. XH: Investigation, Writing – review & editing.
