# Supplementary material for: A study of the psychological mechanisms of job burnout: implications of person–job fit and person–organization fit
Source: Front Psychol. 2024 Aug 2;15:1351032. doi: 10.3389/fpsyg.2024.1351032 (PMC11328536; doi:10.3389/fpsyg.2024.1351032)
Supplement: Supplementary file 3 [file Table_2.DOCX]

# 1. 人-岗匹配/Person-job fit

a. 我感觉自己和这份工作非常匹配

b. 这份工作的要求与我具备的经验、技能和知识相符

c. 单位提供给我的工作环境与我的期望相符

d. 我的性格和气质特征很适合这份工作

**From:** 翁清雄.（2010）.自我职业生涯管理对职业决策质量的作用机制. 管理评论，22（1）: 82-93.

Singh, R., & Greenhaus, J. H. (2004). The relation between career decision-making strategies and person–job fit: A study of job changers. *Journal of Vocational Behavior*, 64(1), 198-221.

# 2. 人-组织匹配

a. 公司的价值观和我自己的价值观很相似

b. 我觉得我的人格特征和公司的形象特征很匹配

c. 公司能满足我的需求

d. 公司和我之间是一个好的搭配

e. 我的价值观和公司的以及公司其他现有员工的价值观都很匹配

f. 我的价值观和公司其他员工的价值观很相符

g. 我认为公司的价值观和“人格”很能反映出我自己的价值观与人格

**From:** 黄莉, 曹国年. (2008). 机构式社会化策略与态度绩效——员工—组织匹配的中介作用. *经济管理*, (21), 93-100.

Cable, D. M., & Judge, T. A. (1996). Person–organization fit, job choice decisions, and organizational entry. Organizational Behavior and Human Decision Processes, 67(3), 294-311.

# 3. 工作压力

a. 我的工作极具压力

b. 工作中很少没有压力的事情

c. 对于我的职业，感到压力巨大。

**From:**

王红丽，张筌钧．被信任的代价：员工感知上级信任、角色负荷、工作压力与情绪耗竭的影响关系研究［Ｊ］．管理世界２０１６，３６（８）：１１０－１２５．

4. 工作倦怠

a. I feel emotionally drained from my work

b. I feel burned out from my work

c. I feel exhausted when I think about having to face another day on the job

d .Working all day is really stressful for me

e .Work makes me feel like I'm breaking down

a 工作让我感觉身心俱疲

b 下班的时候我感觉精疲力尽

c 早餐起床不得不去面对一天的工作时，我感觉非常累

d 整天工作对我来说确实压力很大

e 工作让我有快要奔溃的感觉。

**From:**

Schaufeli, W. B., Leiter, M. P., & Maslach, C. (1996). MBI General Survey. Palo Alto

CA: Consulting Psychologists Press.

李超平、时勘，分配公平与程序公平对工作倦怠的影响，心理学报，2003年第5期，677-684（修订）中国人民大学公共管理学院组织与人力资源研究所李超平联系，email: lichaoping#ruc.edu.cn（发送邮件时，请将#改为@）。
