# Supplementary material for: A study of the psychological mechanisms of job burnout: implications of person–job fit and person–organization fit
Source: Front Psychol. 2024 Aug 2;15:1351032. doi: 10.3389/fpsyg.2024.1351032 (PMC11328536; doi:10.3389/fpsyg.2024.1351032)
Supplement: Supplementary file 4 [file Table_3.docx]

**1 Person**–**job fit**
a. I feel that I am a very good fit for this job.
b. The requirements of this job fit my experience, skills, and knowledge.
c. The work environment provided by the company meets my expectations.
d. My personality and temperament are suitable for this job.

From: Qingxiong Weng. (2010). The mechanism of the effect of self-career [management](coco://sendMessage?ext={"s$wiki_link":"https://m.baike.com/wikiid/8456859744657886019"}&msg=management" \t "https://www.doubao.com/chat/_blank) on the quality of career decision-making. Management Review, 22(1), 82-93.
Singh, R., & Greenhaus, J. H. (2004). The relation between career decision–making strategies and person–job fit: A study of job changers. Journal of Vocational Behavior, 64(1), 198-221.

**2 Person**–**organization fit**
a. The values of the company are very similar to my own values.
b. I feel that my personality traits fit the image traits of the company.
c. The company can meet my needs.
d. There is a good fit between the company and me.
e. My values are very consistent with those of the company and other existing employees.
f. My values are consistent with those of other employees in the company.
g. I think the company's values and "personality" can well reflect my own values and personality.

From: Li Huang, & Guonian Cao. (2008). The mediating effect of institutionalized socialization strategies and attitude performance–the mediating effect of employee-organization fit. Economic [Management](coco://sendMessage?ext={"s$wiki_link":"https://m.baike.com/wikiid/8456859744657886019"}&msg=Management" \t "https://www.doubao.com/chat/_blank), (21), 93-100.
Cable, D. M., & Judge, T. A. (1996). Person–organization fit, job choice decisions, and organizational entry. Organizational Behavior and Human Decision Processes, 67(3), 294-311.

**3 Work pressure**
a. My job is highly stressful.
b. There are rarely no stress in work.
c. I feel tremendous [pressure](coco://sendMessage?ext={"s$wiki_link":"https://m.baike.com/wikiid/321087322904431229"}&msg=pressure" \t "https://www.doubao.com/chat/_blank) for my career.

From:
 Hongli Wang, & Quanjun Zhang. (2016). The cost of being trusted: The impact of employee perceived supervisor trust, role load, work pressure, and emotional exhaustion. Management World, 36(8), 110-125.

**4 Job burnout**
a. I feel emotionally drained from my work.
b. I feel burned out from my work.
c. I feel exhausted when I think about having to face another day on the job.
d. Working all day is really stressful for me.
e. Work makes me feel like I'm breaking down.

From:
Schaufeli, W. B., Leiter, M. P., & Maslach, C. (1996). MBI General Survey. Palo Alto, CA: Consulting Psychologists Press.
Chao-ping Li, &  [Kan](coco://sendMessage?ext={"s$wiki_link":"https://m.baike.com/wikiid/605745648730914718"}&msg=Kan" \t "https://www.doubao.com/chat/_blank) Shi. (2003). The impact of distribution fairness and procedural fairness on job burnout. Acta Psychologica Sinica, 5, 677-684.
